# Lactate Dehydrogenase A or B Knockdown Reduces Lactate Production and Inhibits Breast Cancer Cell Motility *in vitro*

**DOI:** 10.3389/fphar.2021.747001

**Published:** 2021-10-20

**Authors:** Maitham A. Khajah, Sarah Khushaish, Yunus A. Luqmani

**Affiliations:** Faculty of Pharmacy, Kuwait University, Kuwait City, Kuwait

**Keywords:** breast cancer, endocrine resistance, motility, lactate dehydrogenase, lactate

## Abstract

**Background:** Lactate dehydrogenase (LDH) plays an important role in cancer pathogenesis and enhanced expression/activity of this enzyme has been correlated with poor prognosis. In this study we determined the expression profile of LDH-A and B in normal as well as in endocrine-resistant and -responsive breast cancer cells and the effect of their knockdown on LDH activity, lactate production, proliferation and cell motility.

**Methods:** Knockdown experiments were performed using siRNA and shRNA. The expression profile of LDH and signaling molecules was determined using PCR and western blotting. Intracellular LDH activity and extracellular lactate levels were measured by a biochemical assay. Cell motility was determined using wound healing, while proliferation was determined using MTT assay.

**Results:** LDH-A was expressed in all of the tested cell lines, while LDH-B was specifically expressed only in normal and endocrine-resistant breast cancer cells. This was correlated with significantly enhanced LDH activity and lactate production in endocrine resistant breast cancer cells when compared to normal or endocrine responsive cancer cells. LDH-A or -B knockdown significantly reduced LDH activity and lactate production, which led to reduced cell motility. Exogenous lactate supplementation enhanced cell motility co-incident with enhanced phosphorylation of ERK1/2 and reduced E-cadherin expression. Also, LDH-A or -B knockdown reduced ERK 1/2 phosphorylation.

**Conclusion:** Enhanced cell motility in endocrine resistant breast cancer cells is at least in part mediated by enhanced extracellular lactate levels, and LDH inhibition might be a promising therapeutic target to inhibit cancer cell motility.

## Introduction

In normal human tissues a small amount of cellular ATP is derived from glucose breakdown through glycolysis, and the bulk of it by mitochondrial oxidative phosphorylation (mop). During short periods of oxygen insufficiency (hypoxia) such as after intense muscle activity, there may be a temporary shift to anaerobic metabolism that results in pyruvate being shunted into lactate (by action of lactate dehydrogenase; LDHA), which is re-converted back to pyruvate for entry into the previously blocked tricarboxylic acid (TCA) cycle when oxygen levels are restored. Cancer cells however, appear to depend predominantly on glycolysis alone for ATP production even in aerobic conditions [the Warburg effect; (Crozier, 1924; Liberti and Locasale, 2016)] resulting in a build-up of lactate. Despite intense investigation, the reasons for this inefficient glucose utilization remains a mystery. Whereas normal cells produce their ATP through a combination of glycolysis and oxidative phosphorylation, cancer cells mostly dispense with the latter pathway and rely on the production of just two ATP molecules by the conversion of glucose to lactate. Why cancer cells would choose to produce their energy in such an inefficient and apparently wasteful manner has remained a perplexing question, and the subject of extensive debate. Many explanations have been forthcoming. Clearly, conservation of nutrients is not an issue for cancer cells whose main purpose is proliferation, so it is thought that cancer cells adapt in a way that utilizes this shorter pathway to facilitate the formation (possibly through the pentose phosphate shunt) of intermediates for macromolecular biosynthesis which would drive cell replication perhaps at a faster rate., The inefficient ATP production is compensated by excessive usage of glucose at the expense of the host, through increased uptake into the tumour mass facilitated by erratic but extensive neo-angiogenesis. Also, rapidly proliferating cells have important metabolic requirements which extend beyond ATP, such as acetyl-CoA and nicotinamide adenine dinucleotide phosphate (NADP+) (Vander Heiden et al., 2009). A number of excellent reviews (Vander Heiden et al., 2009; Brahimi-Horn et al., 2011; Hay, 2016) discuss these issues in more detail.

In order to avoid cytoplasmic acidosis, cancer cells extrude this excessive lactate from the cell by co-transport with H^+^ through monocarboxylate transporters (Abaza and Luqmani, 2013; Halestrap, 2013). This results in a more acidified environment surrounding the tumour mass as compared with the extracellular matrix around normal tissues. This increased acidification has been proposed to aid in matrix degradation thereby enabling invasion and promoting metastasis (Parolini et al., 2009) as such conditions are thought to favor activity of degradative enzymes that allow cancer cells to move through the matrix. On the other hand, there is some evidence to indicate that acidosis can impede the early stages of carcinogenesis, lead to growth arrest and alter sensitivity to chemotherapeutic drugs (Reichert et al., 2002), while at later stages it correlates with tumour aggressiveness and the acquisition of pro-invasive properties (Abbey et al., 2004). Extensive studies carried out in our laboratory on *in vitro* breast cancer cell models have led us to the conclusion that *alkaline* (as opposed to acidic) pH conditions in the extracellular environment of, particularly endocrine resistant, breast cancer cells actually promotes cell invasion, in part through enhanced matrix metalloproteinase activity (e.g. MMP 2/9) (Khajah et al., 2013; Khajah et al., 2015). In the absence of definitive experiments on tumours *in situ* this apparent contradiction remains to be resolved. Non-uniform distribution of lactate transporters could result in a matrix that is heterogeneous with respect to pH and allow for regions of variable acidity and alkalinity.

LDH is a ubiquitous cytosolic enzyme encoded principally by two genes, LDH-A and LDH-B. Two other genes termed LDH-C and LDH-D (Li, 1990) have also been reported but there is scarce information available on the function of these genes or their contribution to biological activity of the enzyme. The published expression profile of LDH-C in normal tissue is strictly limited to the testis (Goldberg et al., 2010). Low LDH-D expression has been reported in various organs including the kidney and the liver (Wang et al., 2018). The commonly described LDH enzyme is considered to be a tetrameric protein composed of 4 polypeptide chains encoded by either LDH-A or LDH-B giving rise to 5 isoenzymes that are designated as LDH1 (A4), LDH2 (A3B), LDH3 (A2B2), LDH4 (AB3), and LDH5 (B4) exhibiting different kinetic and regulatory properties (Eventoff et al., 1977). As mentioned above, LDH is used by cancer cells to bypass oxidative phosphorylation and produce lactate from pyruvate (Vander Heiden et al., 2009). LDH-A is abundant in skeletal muscle (Miao et al., 2013), while LDH-B is mainly expressed in the heart and brain (Miao et al., 2013). Enhanced expression of LDH isoenzymes has been shown in various tumours such as pancreatic, colorectal and squamous cell head and neck cancer, and correlated with progression and poor clinical prognosis (Markert, 1963; Balinsky et al., 1983; Koukourakis et al., 2005; Koukourakis et al., 2009). Its enhanced expression in tumours results from genetic alteration and hypoxic environment (Gatenby and Gillies, 2004; Pelicano et al., 2006; Levine and Puzio-Kuter, 2010). Enhanced LDH-A activity stimulates ATP generation by reduction of NAD^+^ during glycolysis (Everse and Kaplan, 1973). Some reports suggest the involvement of LDH-B in mTOR-mediated tumorigenesis (Zha et al., 2011) as its expression was found to be up-regulated in stromal cells of human breast cancer samples (Bonuccelli et al., 2010a). A report by Zha et al. (2011) suggested that LDH-B can be transcriptionally activated by STAT3; a downstream component of the growth factor receptor tyrosine kinase cascade initiated through PI3K and mTOR. Treatment of several cancer cell lines with rapamycin was shown to inhibit mTOR-induced STAT3 activation and reduced LDH-B expression. LDH-C was found to be predominantly expressed in the growing testes and spermatozoa (Rodriguez et al., 2003), with no clear relation to cancer pathogenesis. Some reports demonstrated enhanced LDH-C expression in various tumours such as lung, melanoma, kidney, and breast (Koslowski et al., 2002; Kong et al., 2016; Hua et al., 2017; Chen et al., 2021), and being a promising target for cancer therapy (Thomas et al., 2020; Naik and Decock, 2021).

The aim of the current study was to determine the expression profile of LDH-A and B in normal (MCF10A) as well as in endocrine-resistant (pII, MDA-MB-231, and YS2.5) and responsive (MCF7, and YS1.2) breast cancer cells and the effect of their knockdown on LDH activity, lactate production, proliferation and cell motility. We showed that LDH-A was expressed in all of the tested cell lines, while LDH-B was specifically expressed only in normal and endocrine-resistant, but not responsive, breast cancer cells. This was correlated with significantly enhanced LDH activity and lactate production in endocrine resistant breast cancer cells when compared to normal or endocrine responsive cancer cells. Knockdown of either LDH-A or B in breast cancer cells significantly reduced LDH activity and lactate production, which led to reduced cell motility. Culturing the slowly motile YS1.2 in media derived from pII cells (which contain high lactate levels) significantly enhanced their motility. Also, exogenous lactate supplementation to YS1.2 enhanced their motility (but not proliferation); this was accompanied by enhanced ERK1/2 phosphorylation and reduced E-cadherin expression. Also, LDH-A or -B knockdown reduced ERK 1/2 phosphorylation. These data suggest that enhanced cell motility in endocrine resistant breast cancer cells is in part mediated by enhanced extracellular lactate levels, and LDH inhibition might be a promising therapeutic target to inhibit cancer cell motility.

## Materials and Methods

### Cell Lines

MCF10A normal breast epithelial cells were obtained from Dr. E Saunderson and Dr Jenny Gomm St Bartholomews Hospital, London. MCF7 (ER +ve) and MDA-MB-231 (*de novo* ER −ve) breast cancer cells were obtained from the American Type Culture Collection (VA, United States). YS2.5, and pII (*acquired* ER −ve) cell lines were established in our laboratory by transfection of MCF7 with ER directed shRNA plasmid as previously described (Luqmani et al., 2009; Al Saleh et al., 2011). YS1.2 was also derived from MCF7 cells transfected with the shRNA plasmid but failed to downregulate ER and therefore we have used this line as a control for the ER down-regulated lines pII, and YS2.5. For routine culture, all cancer cell lines were maintained as monolayers in advanced Dulbecco’s minimum essential medium (DMEM) containing phenol red and supplemented with 5% fetal bovine serum (FBS), 600 μg/ml L-glutamine, 100 U/ml penicillin, 100 μg/ml streptomycin and 6 ml/500 ml 100 × non-essential amino acids (all from Invitrogen, CA, United States), and were grown at 37°C in an incubator gassed with an atmosphere of 5% CO_2_ and maintained at 95% humidity. MCF10A were cultured in DMEM F12 (Cytiva, Cat# SH30023.01) supplemented with 5% horse serum, 1x Pen/Strep, 20 ng/ml mouse EGF, 0.5 μg/ml hydrocortisone, 100 ng/ml cholera toxin and 10 μg/ml insulin.

### RNA Extraction

RNA was extracted from cells and purified using the RNeasy Kit (Qiagen, Cat # 79254) according to the manufacturer’s protocol. The concentration and yield of RNA was determined spectroscopically using the Nano-Drop (Pharmacia) and integrity was checked by gel electrophoresis.

### Quantitative Real Time PCR

RNA was converted to cDNA using a High-Capacity cDNA Reverse Transcription Kit from Applied Biosystems (Cat# 10400745). Taqman real time quantitative PCR was used to measure expression of LDH-A, -B, -C- and -D target genes using proprietary primer/probe mixes from the manufacturer (OriGene, United States) [Cat# LDH-A (HP208683), LDH-B (HK204305), LDH-C (HK210517), and LDH-D (HP218158)]. A master reaction mix was prepared for the appropriate number of samples and 23 µL was pipetted into an ABI optical 96-well plate and 2 µL of cDNA sample was added to each well. The plate was then placed into a 7500 HT fast real-time PCR thermocycler and amplification performed according to the standard procedure given in the ABI Taqman manual, and was the same for all primers. Control gene (human actin) was labelled with “JOE” and target genes with “FAM”.

### Western Blotting

Cells were cultured in 6 well plates to 80–90% confluence. The medium was subsequently aspirated off and cell monolayers harvested by scraping and re-suspension into 300 μL of lysis buffer containing 50 mM HEPES, 50 mM NaCl, 5 mM EDTA 1% Triton X, 100 μg/ml phenylmethylsulfonyl fluoride (PMSF), 10 μg/ml aprotinin and 10 μg/ml leupeptin. This was transferred into an Eppendorf tube and stored at −80°C. Protein concentration was determined by the Bradford assay using BSA as standard; 8 μg protein lysate was mixed with an equal volume of 2 × SDS and heated at 90°C for 10 min. Samples were loaded onto a 10% SDS-polyacrylamide gel and electrophoresed at 150 V for 1 h. Proteins were transferred to a nitrocellulose membrane and blocked with 2% BSA for 1 h before being incubated overnight at 4°C with primary antibodies (prepared in 2% BSA) against actin (loading control, Cell signaling, United States, 1:1000 dilution, Cat # 4970), phospho- to total-LDH-A (Cat # 8176, 3582), p38 MAPK (Cat # 9212), AKT (Cat # 9271), ERK1/2 (Cat # 4695), Focal adhesion kinase (Cat # 3285), and E-cadherin (Cat # 3195) (Cell signaling, United States, 1:1000 dilution), total-LDH-B (Cat # SAB1404017), LDH-C (Cat # SAB1402835), and LDH-D (Cat # ABC927) (Sigma-Aldrich, 1: 1000 dilution). The membrane was then washed and incubated with anti-HRP-conjugated secondary antibody (1/500 dilution) (Cell signaling, United States Cat # 7074) for 1 h developed with Super Signal ECL (Thermo Scientific, Rockford, United States) and visualized with a Cell bio-imager (ChemiDoc MP System from BioRad, United States).

### Lactate Assay

Cells were cultured to a density of approximately 10^6^ in 6-well microtiter plates. The culture medium was aspirated into Eppendorf tubes and protein concentration was estimated using the Bradford assay. Extracellular lactate was measured in aliquots using the EnzyChrom L-Lactate Assay Kit ECLC-100 (BioAssay Systems United States), following the manufacturer’s protocol. Standards were prepared by dilution of a stock solution of 100 mM L-lactate in serum free media, and 20 μL of samples or standards were transferred into wells of a clear bottom 96-well plate. Two reactions were performed for each sample: one with both enzymes A and B, and another without enzyme A (control). The working reagent was prepared freshly by mixing 60 μL Assay Buffer, 1 μL enzyme A, 1 μL enzyme B, 10 μL NAD and 14 μL MTT. For control, enzyme A was omitted from the reagent mix; 80 μL of the working reagent was added to each sample well and mixed by pipetting up and down. The background optical density at 650 was measured in a plate reader at “zero” time (OD_0_) and after 20 min (OD_20_) incubation at room temperature and subtracted from that at 565 nm. For the standard curve the corrected OD_0_ was subtracted from OD_20_. For samples with no enzyme A control, the ΔOD _no enzA_ value was subtracted from ΔOD _sample_. The ΔΔOD values were used to determine sample L-lactate concentration from the standard curve.

### Lactate Dehydrogenase Assay

Quantichrom Lactate Dehydrogenase Kit (DLDH-100; BioAssay Systems) was used following the manufacturer’s protocol for the same samples that were prepared for the lactate assay. Freshly prepared assay reagent, containing 14 μL MTT solution, 8 μL NAD solution, and 170 μL Substrate buffer, were aliquoted into wells of a 96-well plate at room temperature and 10 μL of each sample was added to start the reaction. Control wells contained 200 μL of H_2_O (for OD_H2O_) and 200 μL of Calibrator (for OD_Cal_). The absorbance of the solution at 565 nm was determined in a plate reader spectrophotometer at zero time and again after 25 min (OD_25_). LDH activity was calculated according to the equations provided in the protocol. As explained above for lactate, the intracellular LDH activity is expressed as a percentage of the control (or one cell line versus another) rather than in absolute units.

### siRNA-Mediated Knockdown of LDH-A or -B mRNA

Cells were seeded into 12-well plates and cultured for 24 h to reach 60–80% confluency before transfection using Lipofeactamine RNAiMAX reagent (Santa Cruz, Cat # sc-43893, sc-45899). The procedure was performed according to the manufacturer’s protocol. In brief, solution A (100 μL of optiMEM + 6 μL reagent), and solution B (100 μL of optiMEM + 20 pmol of siRNA) was prepared and mixed gently and incubated for 15 min at room temperature. For each well, this complex was added drop-wise to the wells containing 1 ml DMEM media, and placed in a 37°C, 5% CO_2_ incubator for 48 and 72 h. Knockdown of LDH A, B, C and D mRNA was confirmed by western blotting and qPCR. The raw threshold cycle (CT) values was analyzed by the ΔΔCt method using the spreadsheet developed by Pfaffl to determine normalized expression ratios of target genes (Bustin et al., 2005).

### LDH-A and -B mRNA Knockdown Using Lentivirus

For the shRNA transfection, cells were seeded into 12 well plate to approximately 50% confluency. On the following day, 1 ml DMEM medium containing 5 μg/ml polybrene was added plus 10 µL of lentiviral particles containing shRNA directed against LDHA or LDHB, gently swirled, and incubated for 24 h (Santa Cruz, Cat # sc-45899-V, sc-43893-V). After that, the medium was changed with fresh DMEM medium minus polyberene and incubated for another 24 h. The following day, cells were sub-cultured 1:5 and incubated for a further 24–48 h. Finally, DMEM media containing fresh puromycin (2 μg/ml) was added for selection of transformants; medium containing fresh antibiotics was replaced every 3 days until defined resistant colonies could be selected, expanded and tested using western blotting analysis to confirm the knockdown.

### Cell Motility Assay

Cells were cultured in 6 well plates with complete DMEM to 80–90% confluence. A scratch was created in the cell monolayer using a sterile p1000 pipette tip and a photograph of the scratched area was taken immediately (0 h). The plates were then placed in a 37°C, 5% CO_2_ incubator. After overnight incubation, another photograph was taken of the same scratched area. The width of the scratch at 24 h was calculated as a percentage of the width at 0 h; a minimum of three areas along the scratch was measured.

### MTT Assay

Cells were routinely seeded into 24-well culture plates and allowed to grow to 30–35% confluency. Cell density was determined either immediately (day zero) or after 1 and 4 days of cultivation. For the measurement, medium was removed and replaced with 500 µL of MTT reagent (Sigma-Aldrich, United States) (0.5 mg/ml) and left at 37°C for 2 h; MTT solution was removed and 200 µL of acidic isopropanol added to dissolve the blue formazan crystals that had formed. Plates were scanned at 595 and 650 nm (for background subtraction) using a MULTISKAN SPECTRUM spectrophotometer, and absorbance compared between samples as a measure of proliferation.

### Oxygen Consumption

Cells were seeded to 80–90% confluency, then trypzinized, centrifuged and resuspended in DMEM medium to 5 × 10^6^ cell/ml. Using YSI model 533 biological oxygen monitor machine, oxygen consumption was measured. Samples were loaded in the corresponding chambers with an equal amount of medium, then electrodes were inserted for reading, recordings were taken for 10 min. For data analysis; on the horizontal scale, 1 cm = 50 s, i. e 10 min = 12 cm. The speed of the chart was 0.2 mm/s. On the vertical scale, the whole distance from 0 to 100 corresponds to 0.265 umol/ml O_2_ consumed, therefore, ΔO_2_/10 min is measured from the recorded graph. Then all results were calculated as %O_2_ consumed per ml for 10 min (12 cm) $\frac{\text{Cell number}/\text{ml}}{\text{ Chamber Vol}.\;\left(\text{ml}\right)}$ .To calculate the O_2_ consumed as nmol/ml/10 min the value obtained from the % O_2_ was multiplied by 2.65 (1% decrease of O_2_ = 2.65 nmol/ml). Finally, nmol Oxygen consumed/10^6^ cell/10 min was calculated by using this equation $\frac{\left[\left(\Delta \text{O}2/10\text{ min}\right)\text{*}2.65\text{ nmol}/\text{ml}\right]}{\left[\left(\text{Cell number}/\text{ml}\right)/\text{Chamber Vol}.\text{ ml}\right]}$ .

### Statistical Analysis

Means of experimental groups were compared with controls using the student’s t-test or one-way ANOVA followed by Bonferroni post-hoc test. Statistical significance was assumed at *p* values < 0.05 using GraphPad Instat software. GraphPad Prism 6 was used to plot graphs.

## Results

### Expression Profile of LDH-A and -B, LDH Activity, and Lactate Levels in Normal Breast Epithelial Cells and in Endocrine-Sensitive and -Resistant Breast Cancer Cell Lines

**FIGURE 1:**
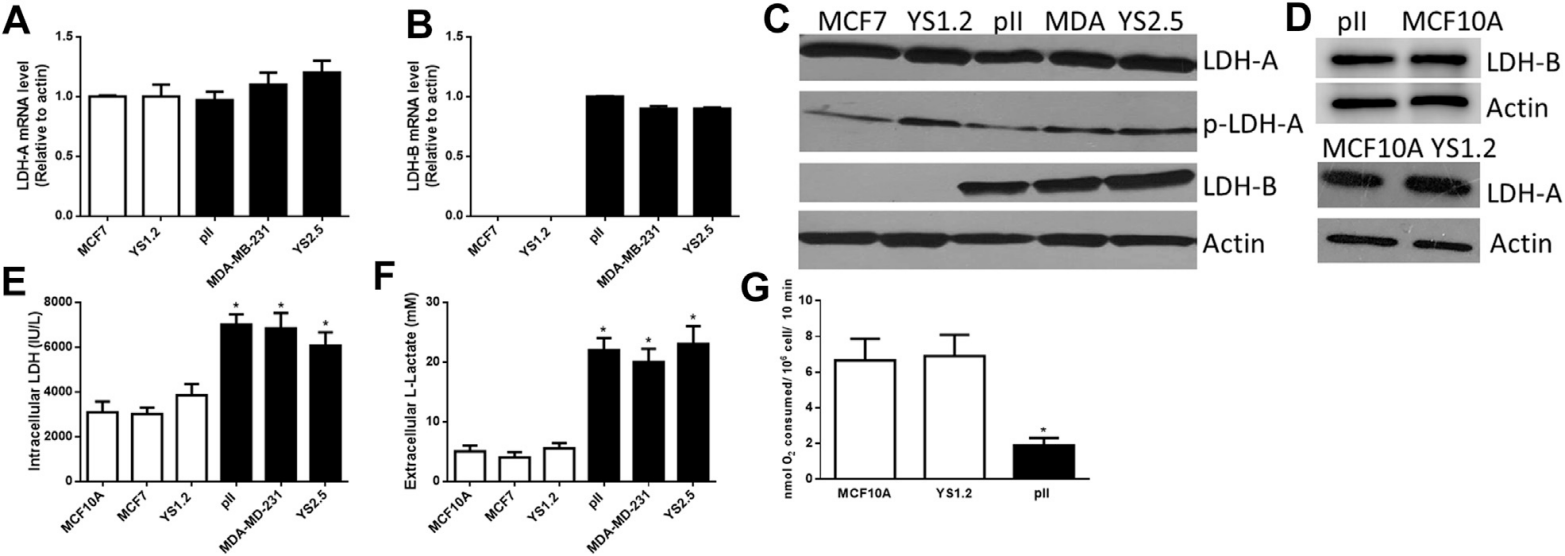
The expression profile of lactate dehydrogenase isoenzymes and lactate in the tested cell lines. LDH-A [**(A)**, normalized to MCF7], and LDH-B [**(B)**, normalized to pII] mRNA expression profile in ER +ve (MCF7 and YS1.2) and ER −ve (pII, MDA-231, and YS2.5) cells. *p < 0.05 Vs. MCF7 and YS1.2 (*n* = 3 per group). **(C,D)** show western blotting analysis of LDH-A (total and phospho), LDH-B, and actin (loading control) for the tested cell lines. The blot represents one out of three similar experiments. **(E,F)** show intracellular LDH activity and extracellular lactate level in the tested cell lines. *p < 0.05 Vs. YS1.2, MCF7, and MCF10A (*n* = 6 per group). **(G)** shows oxygen consumption rate in the tested cell lines. *p < 0.05 Vs. YS1.2, MCF10A (*n* = 6 per group).

Figures 1A,B shows differential expression of LDH-A and LDH-B mRNA in various breast cancer cell lines; LDH-A was expressed in both ER +ve (MCF7 and YS1.2) and ER −ve lines (MDA-MB-231, pII and YS2.5), while LDH-B was specifically expressed only in ER −ve cells. This was also confirmed at protein level using western blotting analysis (Figure 1C); total/phospho-LDH-A was expressed in both ER +ve and –ve lines, while LDH-B was only expressed in ER −ve lines. The normal breast epithelial cell line MCF10A expressed LDH-A and -B isoenzymes (Figure 1D). We were unable to detect LDH-C or -D mRNA or protein in the tested cell lines (data not shown).

LDH activity was predominantly detected inside the cell with little activity found in the culture medium; and this is also most likely due to release from lysed dead cells in the culture medium. Lactate was predominantly detected outside rather than inside the cells. We observed significantly higher LDH activity and lactate levels in ER −ve (pII, MDA-MB-231, and YS2.5) compared to ER +ve (MCF7 and YS1.2) breast cancer cells (Figures 1E,F). As shown in Figure 1G, reduced oxygen consumption (i.e. enhanced anaerobic activity) was observed in ER −ve breast cancer cells when compared to ER +ve breast cancer cells or normal breast epithelial cells, which is consistent with the higher LDH activity and lactate levels in ER −ve (pII) cells. Having established the consistency of our data using several cell lines, in the subsequent experiments we studied the effect of LDH isoenzyme knockdown using one representative ER −ve (pII) and one ER +ve (YS1.2) breast cancer cell line.

### Effect of LDH-A or -B Knockdown on Breast Cancer Cell Motility

**FIGURE 2:**
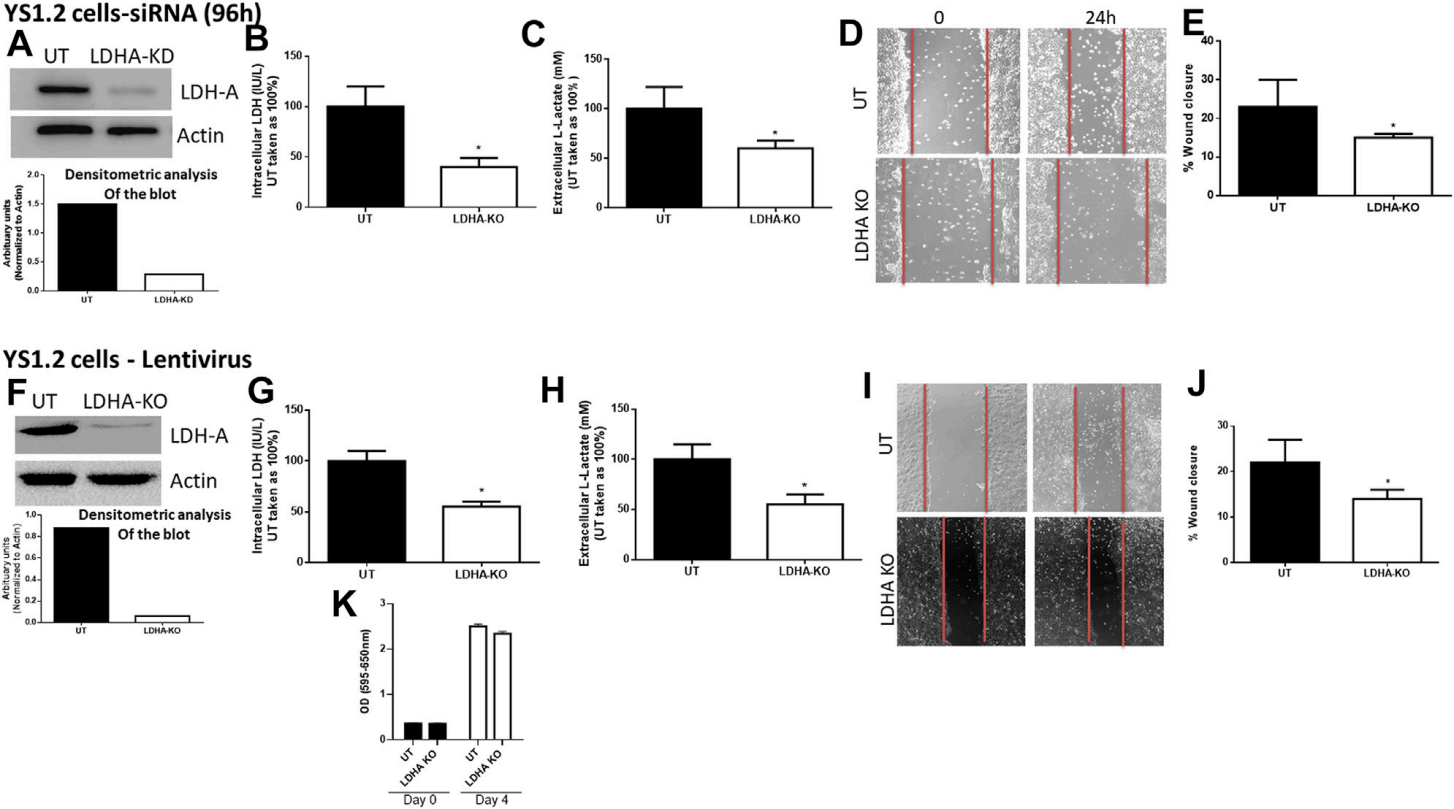
Effect of LDH-A knockdown in the ER +ve breast cancer cell YS1.2. **(A)** show western blotting analysis of LDH-A and actin (loading control) in untreated (UT), or cells treated with LDH-A siRNA for 96 h. Densitometric analysis of the blot is also included. The blot represents one out of three similar experiments. **(B,C)** show LDH activity and lactate level respectively. *p < 0.05 Vs. UT cells (*n* = 3 per group). **(D,E)** show the percentage of wound closure (using scratch assay). *p < 0.05 Vs. UT cells (*n* = 6 per group). **(F)** show western blotting analysis of LDH-A and actin (loading control) in untreated (UT), or cells treated with LDH-A lentivirus. Densitometric analysis of the blot is also included. The blot represents one out of three similar experiments. **(G,H)** show LDH activity and lactate level respectively. *p < 0.05 Vs. UT cells (*n* = 3 per group). **(I,J)** show the percentage of wound closure (using scratch assay). *p < 0.05 Vs. UT cells (*n* = 6 per group). **(K)** show cell proliferation (using MTT assay) at day 0 (seeding day) and day 4 (*n* = 3 per group).

First, we determined the effect of LDH-A knockdown in YS1.2 and pII cells. As shown in Figure 2, LDH-A expression was significantly decreased in siRNA-treated YS1.2 (Figure 2A) and this leads to significant reduction (by 50%) in total LDH activity (Figure 2B), extracellular lactate level (Figure 2C), and cell motility (Figures 2D,E). It should be noted that LDH-B isoenzyme was not expressed in YS1.2 cells; either UT or LDHA-knockdown (KD, data not shown). This was also shown using lentiviral mediated transfection against LDH-A in YS1.2 cells (Figures 2F–I). LDH-A knockdown did not change YS1.2 proliferations when compared to untreated cells (Figure 2K). It should be noted that LDH-B isoenzyme was not expressed in YS1.2 cells; either UT or LDHA-knockout (KO, data not shown).

**FIGURE 3:**
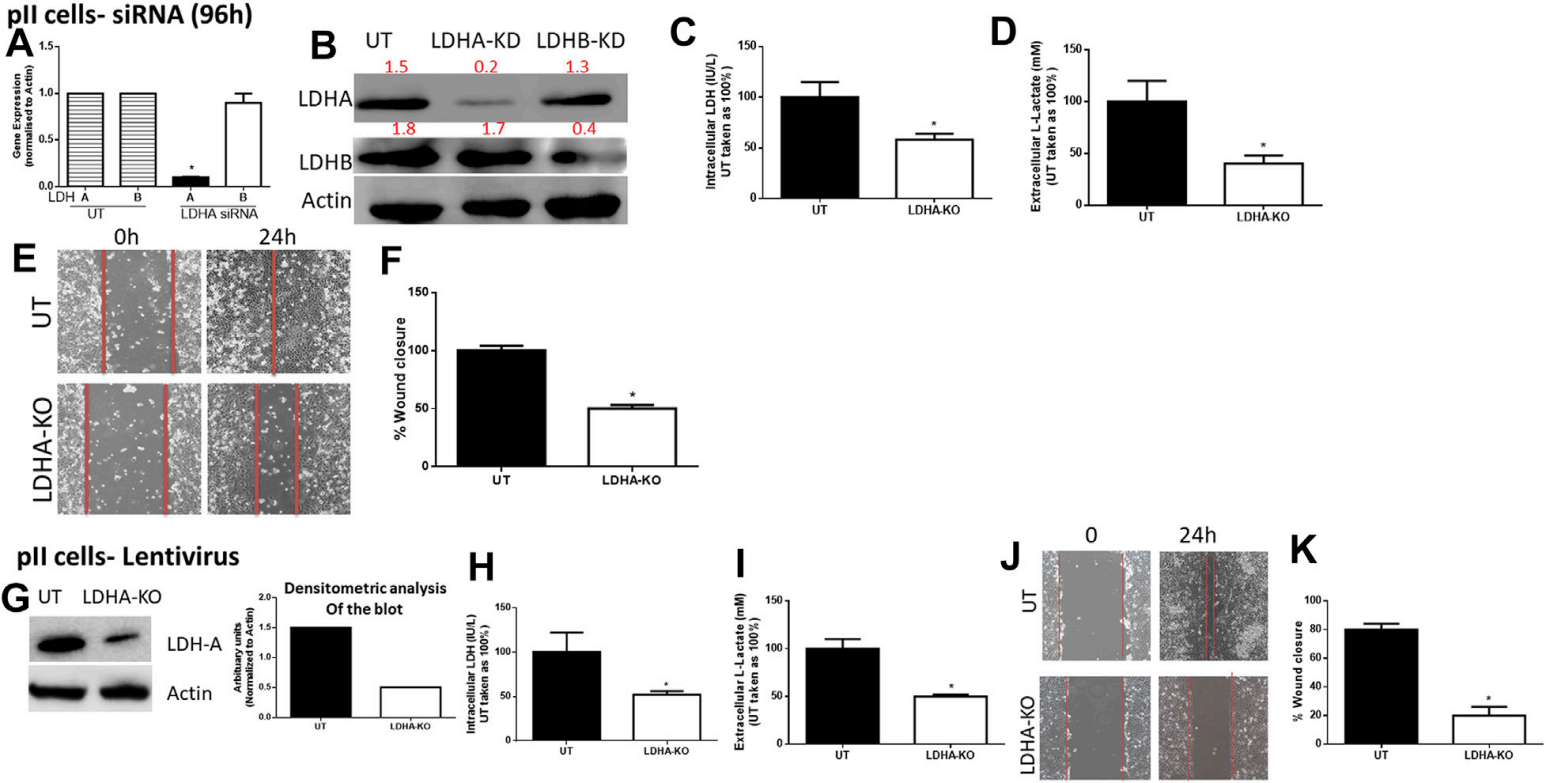
Effect of LDH-A knockdown in the ER −ve breast cancer cell pII, **(A)** show LDH-A and LDH-B (normalized to actin) mRNA expression profile. *p < 0.05 Vs. UT cells (*n* = 3 per group). **(B)** show western blotting analysis of LDH-A and actin (loading control) in untreated (UT), or cells treated with LDH-A or LDH-B siRNA for 96 h. Densitometric analysis of the blot is also included. The blot represents one out of three similar experiments. **(C,D)** show LDH activity and lactate level respectively. *p < 0.05 Vs. UT cells (*n* = 3 per group). **(E,F)** show the percentage of wound closure (using scratch assay). *p < 0.05 Vs. UT cells (*n* = 6 per group). **(G)** show western blotting analysis of LDH-A and actin (loading control) in untreated (UT), or cells treated with LDH-A lentivirus. Densitometric analysis of the blot is also included. The blot represents one out of three similar experiments. **(H,I)** show LDH activity and lactate level respectively. *p < 0.05 Vs. UT cells (*n* = 3 per group). Panels **(J,K)** show the percentage of wound closure (using scratch assay). *p < 0.05 Vs. UT cells (*n* = 6 per group).

For pII cells (Figure 3), LDH-A (but not LDH-B) mRNA (Figure 3A) and protein (Figure 3B) expression was significantly decreased in LDH-A-siRNA-treated cells. We did not see evidence of any compensatory increase in LDH-B expression when LDH-A gene expression was reduced. LDH-A knockdown led to a significant reduction (by 50%) in total LDH activity (Figure 3C), lactate level (Figure 3D), and cell motility (Figures 3E,F). This was also seen when using lentiviral mediated transfection against LDH-A in pII (Figures 3G–K).

**FIGURE 4:**
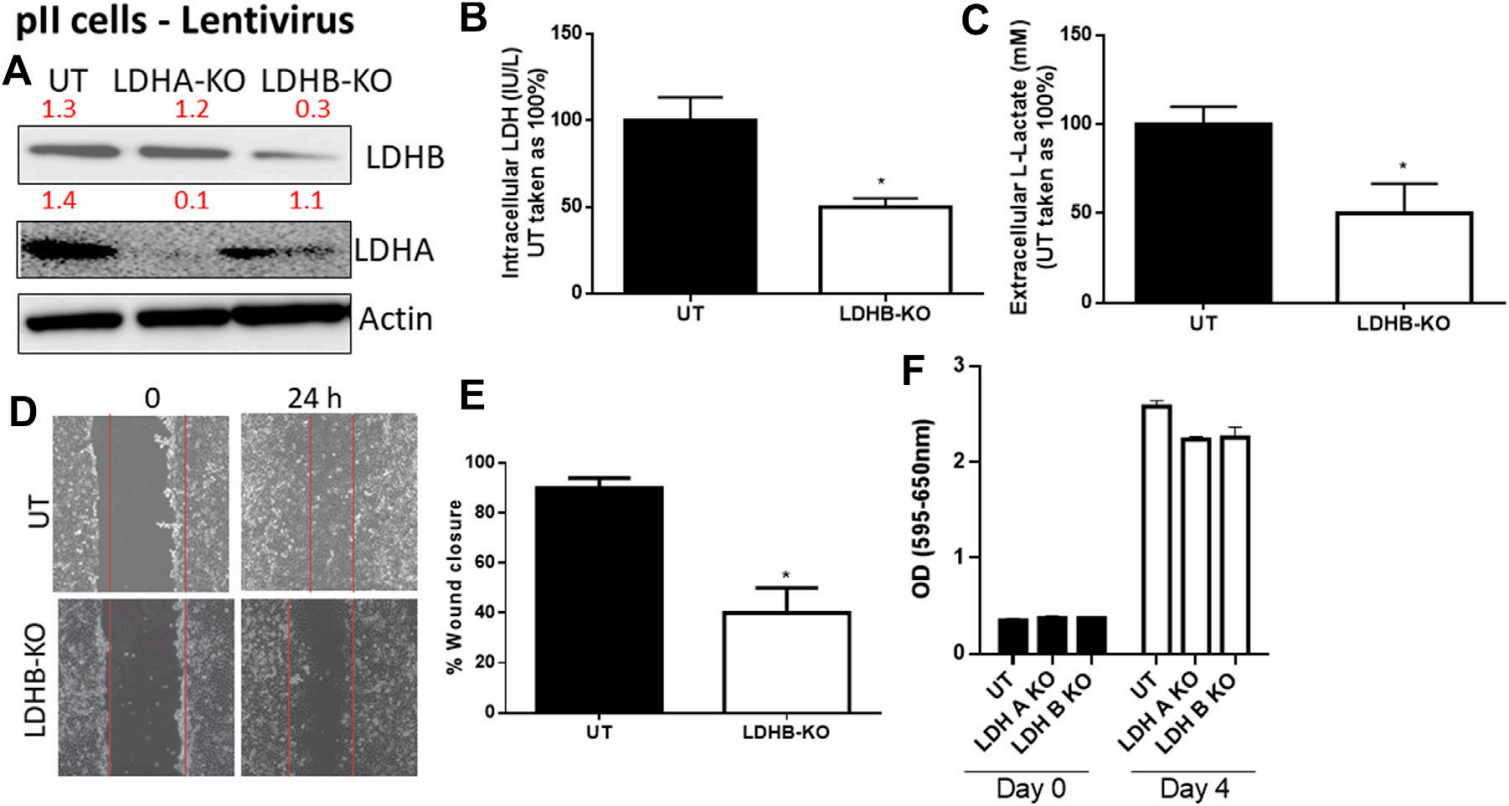
Effect of LDH-B knockdown in the ER −ve breast cancer cell pII. **(A)** show western blotting analysis of LDH-B and actin (loading control) in untreated (UT), or cells treated with LDH-A or -B lentivirus. Densitometric analysis of the blot is also included. The blot represents one out of three similar experiments. **(B,C)** show LDH activity and lactate level respectively. *p < 0.05 Vs. UT cells (*n* = 3 per group). **(D,E)** show the percentage of wound closure (using scratch assay). *p < 0.05 Vs. UT cells (*n* = 6 per group). **(F)** show cell proliferation (using MTT assay) at day 0 (seeding day) and day 4 (*n* = 3 per group).

We next determined the effect of LDH-B knockdown in pII cells (note: YS1.2 cells do not express LDH-B). As shown in Figure 4, LDH-B expression was significantly decreased in lentivirus-treated when compared to untreated cells (Figure 4A). We did not see evidence of any compensatory increase in LDH-A expression when LDH-B gene expression was reduced. This led to a significant reduction (by 50%) in total LDH activity (Figure 4B), lactate levels (Figure 4C), and cell motility (Figures 4D,E). As shown in panel F, LDH-A or -B knockdown did not modulate pII proliferative activity when compared to untreated cells.

### Effect of Lactate on Breast Cancer Cell Motility

**FIGURE 5:**
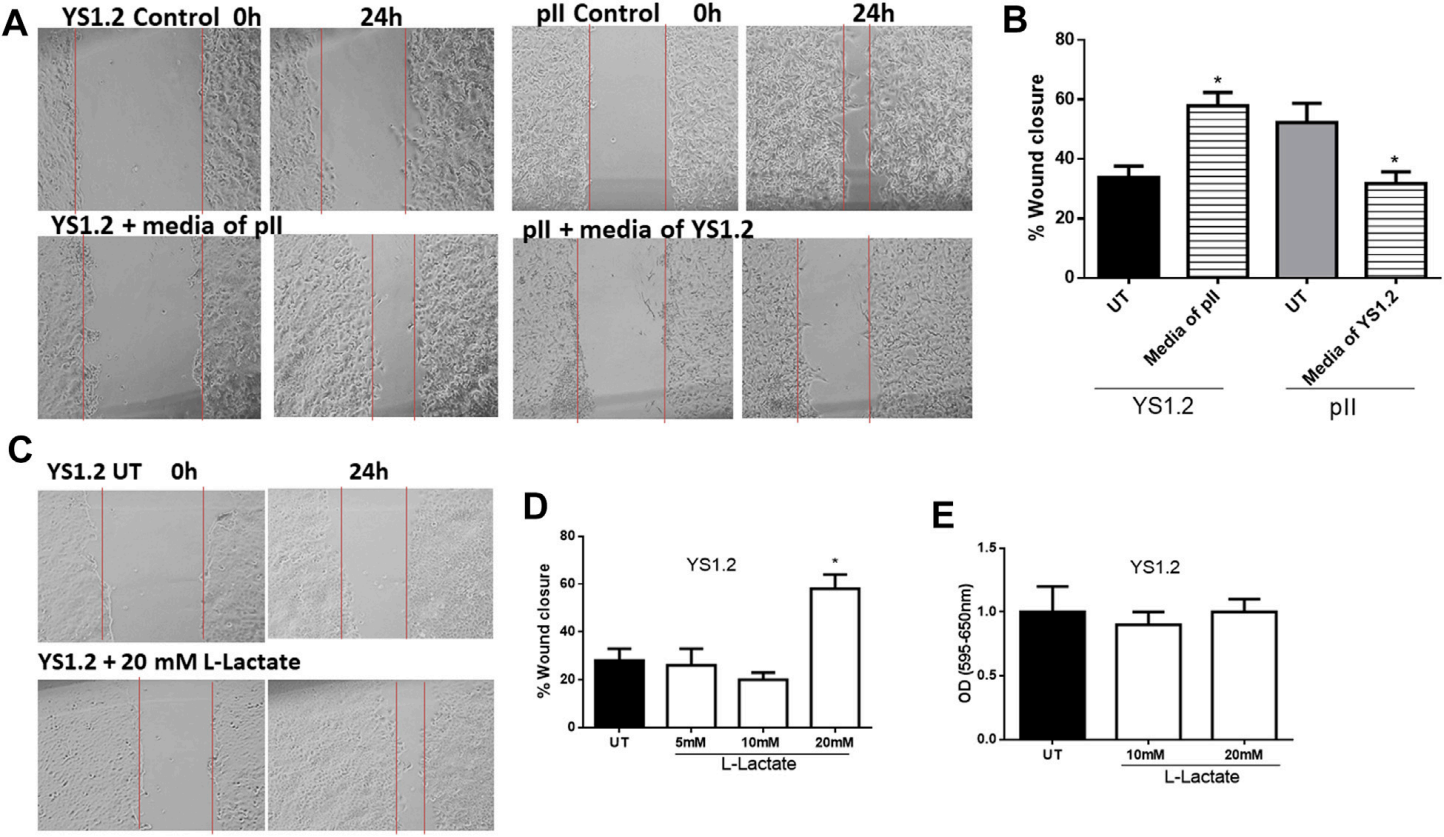
Effect of lactate supplementation on breast cancer cell motility. **(A–D)** show the percentage of wound closure (using scratch assay) in pII or YS1.2 using different treatment approaches. *p < 0.05 Vs. UT cells (*n* = 6 per group). **(E)** show cell proliferation (using MTT assay) at day 4 (*n* = 3 per group).

We previously showed that pII cells secrete more lactate into the extracellular environment when compared to MCF10A or YS1.2 (Figure 1). As shown in Figures 5A,B, culturing YS1.2 (for 24 h) with conditioned media aspirated from a culture of pII cells, significantly enhanced cell motility. On the other hand, culturing pII cells with conditioned media derived from a culture of YS1.2 cells significantly reduced cell motility. We hypothesized that this difference might be due to the higher amount of lactate present in the culture medium derived from pII cells. To test this notion of a direct effect of lactate on cell motility, we cultured YS1.2 (for 24 h) with increasing amounts of lactate added to the cultivation medium. We observed that at 20 mM lactate [which is similar to the concentration measured in the culture medium of pII cells (Figure 1F) there was significantly enhanced cell motility (Figures 5C,D) but not proliferation (Figure 5E).

**FIGURE 6:**
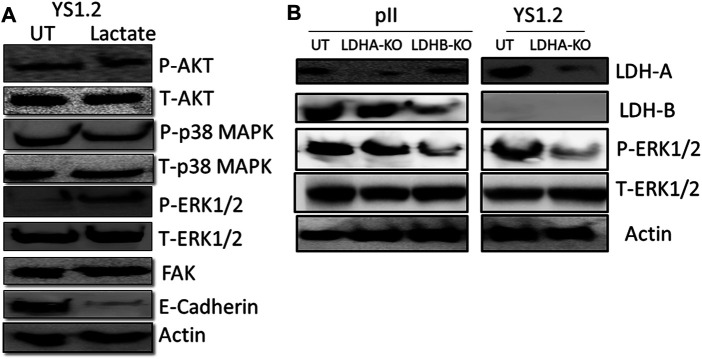
Effect of lactate supplementation or LDH isoenzyme knockdown on the expression/activity of signaling molecules. **(A)** shows western blotting analysis of P-AKT, P-p38 MAPK, P- and T-ERK1/2, FAK, E-cadherin, and actin (loading control) in YS1.2 either untreated (UT), or cells treated with lactate (20 mM for 24 h). The blot represents one out of three similar experiments. **(B)** shows western blotting analysis of LDH-A, LDH-B, P-ERK 1/2, and actin (loading control) in pII or YS1.2 cells, either untreated (UT) or treated with LDH-A or -B lentivirus. The blot represents one out of three similar experiments.

We also did a preliminary investigation of some major signaling molecules to see if these may be affected. Western blotting of lysates of YS1.2 cells treated with 20 mM lactate had increased phosphorylation of ERK1/2 but did not show any change in either p38 MAPK or AKT phosphorylation (Figure 6A). Also, the expression profile of focal adhesion kinase (FAK) was not modulated by lactate treatment. Interestingly, E-cadherin expression was significantly reduced by lactate treatment which might lead to loose cell-cell connection and enhanced degree of motility. Also, LDH-A or -B knockdown in pII and YS1.2 cells reduced ERK1/2 phosphorylation (Figure 6B).

## Discussion

In our laboratory, we have established several *acquired* forms of endocrine resistant breast cancer cell lines by shRNA-mediated silencing of the ER in MCF7 cells (pII, and YS 2.5) (Luqmani et al., 2009). These ER −ve cells have a modified gene expression profile indicating an epithelial to mesenchymal transition (EMT) manifested by reduced expression of epithelial markers (such as E-cadherin) and enhanced expression of mesenchymal markers (such as vimentin). These cells exhibit more aggressive behaviour with enhanced proliferation, motility and invasive properties (Al Saleh et al., 2011; Khajah et al., 2012).

In the present study, we have utilized these cell lines and shown that the acquired ER −ve cells as well as normal MCF10A express both LDHA and LDHB whereas expression of LDHB is lost in ER +ve breast cancer cells. This results in a significant increase in total LDH activity, lactate production, and cell motility in the ER −ve cells. LDH-A knockdown, by RNA interference with either liposome encapsulated siRNA or with shRNA in lentiviral particles, significantly reduced total LDH activity, lactate production, and cell motility in both ER −ve and +ve cells. Experiments with conditioned media indicated that constituents released from pII cells could confer significantly increased motile ability on YS1.2 cells, without any effect on their proliferation. This effect could be mimicked by the addition of lactate to the culture media of YS1.2 cells. Our current data suggest a hitherto un-recognised role of lactate in enhancing cell motility, which may, at least in part, involve signaling through enhanced ERK1/2 phosphorylation and reduced expression of a major adhesion molecule; E-cadherin. Also, LDH-A or -B knockdown in pII and YS1.2 cells reduced ERK1/2 phosphorylation. Thus, targeting LDH activity might be an effective measure in the treatment of breast cancer, particularly after development of endocrine resistance.

Several lines of evidence suggest a direct correlation between the activity of various LDH isoenzymes and advanced stage/prognosis of breast cancer patients. A genome-wide analysis of breast cancer transcriptomes (Charafe-Jauffret et al., 2006) has produced profiles representing a strong lactic acidosis response signature claiming to identify a sub-group of low-risk patients with distinct metabolic profiles suggestive of a preference for anaerobic respiration. This includes repression of glycolytic gene expression and down-regulation of AKT. In a study on Russian patients, LDH activity was analyzed in the serum, primary tumour and adjacent un-involved breast tissue from patients with adenocarcinoma and benign adenofibroma. The LDH activity was increased in both cancerous and adjacent tissues, and its serum level reflected cell membrane alterations in the tumour mass and the surrounding healthy tissue (Shatova et al., 1999). This was confirmed by a recent study showing a direct correlation between high serum LDH activity and high TNM staging in breast cancer patients (Agrawal et al., 2016), including those with a triple negative hormone receptor status (Chen et al., 2016). Furthermore, in a study performed in women with bone metastases from breast cancer, enhanced LDH levels were correlated with a six-fold increased risk of mortality (Brown et al., 2012). In regard to certain LDH genes, one report suggested that LDH-A expression correlated significantly with tumour size in breast cancer patients (Wang et al., 2012). LDH-B was also found to be highly expressed in stromal cells of malignant human breast cancer samples suggesting a role in breast tumourigenesis (Bonuccelli et al., 2010a). Interestingly, Dennison et al. (2013) found a predictive response of LDH-B expression to neoadjuvant chemotherapy, which supports the clinical evolution of LDH-B as a marker of response for breast cancer patients receiving neaoadjuvant chemotherapy. In this report, they also demonstrated (using 2 public mRNA microarray databases and various cell lines) enhanced LDH-B expression in basal-like or triple-negative breast cancer cells and cell lines with lower ER expression profile while LDH-A expression was not different between the various tested cells. Furthermore, there are discrepancies in the literature regarding the specific expression profile of LDH isoforms in breast cancer cells. In one report (Hussien and Brooks, 2011), the authors assayed LDH-A and B in the normal non-transformed human mammary cell line HMEC 184, and compared this with the ER −ve MDA-MB-231 and the ER+ve MCF7 breast cancer cell lines. They reported that the LDH-A isoform was mainly expressed in MDA-MB-231 while MCF7 cells expressed mainly the LDH-B form. This contradicts earlier reports of the absence of LDH-B protein in MCF7 (Balinsky et al., 1983; Dennison et al., 2013). Their results also differ from another report of Charafe-Jauffret et al. (2006) who did not observe any significant change in LDH-A mRNA but instead observed a reduction in LDH-B expression. Similarly, the expression of LDH-A was observed in another study in both MDA-MB-435 and MCF7, and was increased by ectopic expression of c-erbB2 in both lines, whereas the expression of LDH-B was limited only to MDA-MB-435 and was insensitive to transfected c-erbB2 over-expression. No LDH-B mRNA was detectable in MCF7 with or without c-erbB2 over-expression (Zhao et al., 2009). An interesting additional observation was that the high expression of LDH-B was seen in the mesenchymal compared with luminal-like cell lines (Charafe-Jauffret et al., 2006; Dennison et al., 2013; Odet et al., 2013). These data are in agreement with our data where we have shown that LDH-A is expressed in both ER +ve and −ve breast cancer cells while LDH-B is specifically expressed in ER −ve cells where this contributed to enhanced total LDH activity and lactate production (Figure 1).

Enhanced metabolic state (determined by the rate of oxygen consumption and extracellular acidification) was observed in cells with high LDH-B expression profile, but interestingly knocking-down LDH-A or LDH-B mRNA in breast cancer cells resulted in enhanced metabolic rate suggesting a metabolic compensation from knocking down one of the isoenzymes (Dennison et al., 2013). In our report, we showed reduced oxygen consumption rate in ER −ve when compared to ER +ve or normal breast epithelial cells (Figure 1G), suggesting enhanced anaerobic activity in endocrine resistant breast cancer cells which we previously shown to be correlated with enhanced hypoxia inducible factor (HIF-1α) levels (Barrak et al., 2020).

The knockdown of specific isoforms of LDH isoenzymes resulted in different responses in breast cancer cells. In some reports, LDH-A knockdown resulted in reduced cell proliferation, migration and invasion (Wang et al., 2012; Rizwan et al., 2013), while in others, LDH-B knockdown reduced cell metastasis *in vivo* (Zha et al., 2011). In contrast, other reports have demonstrated that knocking down either LDH-A or LDH-B did not modulate cell proliferation or motility (Valvona and Fillmore, 2018), and actually enhanced (rather than reduced) lactate levels (Dennison et al., 2013). Thus neither the role of a specific LDH isoenzyme nor the direct role of lactate (independently of the co-transported H^+^ which is what presumably influences the extracellular pH) has been properly addressed in breast cancer pathogenesis. Also, there might be a compensatory mechanism of the other LDH isoforms when knocking down one of them. shRNA-mediated silencing of LDH-A in MCF7 (ER +ve) and MDA-MB-231 (ER −ve) cell lines resulted in inhibition of cell proliferation (through increased intracellular oxidative stress activity and apoptosis machinery) *in vitro*, and reduced tumourigenic ability *in vivo* (Wang et al., 2012). This was also confirmed using a metastatic breast cancer cell line (4T1), where shRNA-mediated silencing of LDH-A significantly inhibited cell proliferation, migration and invasion both *in vitro* and *in vivo* (Rizwan et al., 2013). In addition, attenuation of LDH-B expression in tumour cells injected into nude mice reduced cell metastasis, in part through reduced activity of the oncogenic mTOR pathway (Zha et al., 2011). Interestingly, in one report using medulloblastoma cells, LDH-A knockdown did not modulate lactate levels, cell proliferation or motility, suggesting the involvement and/or compensation of other isoenzymes (such as LDH-B) responsible for lactate production (Valvona and Fillmore, 2018). Herein, we showed that both LDH-A and -B play an important role in breast cancer motility. LDH-A knockdown in YS1.2 or pII, or LDH-B knockdown in pII cells resulted in significant reduction in total LDH activity, lactate levels, and cell motility, but not proliferation (Figures 2–4). Also, knocking-down one LDH isoenzyme did not lead to a compensatory increased expression profile of the other one (Figures 3A,B, 4A). We also tried to knockdown (using lentivirus) both LDH-A and -B simultaneously in pII cells but this lead to cell death without any colonies formed upon antibiotic selection (data not shown). Also, we were unable to detect LDH-C or -D in our cell lines either by PCR or western blotting (data not shown). In one report using human colon adenocarcinoma and murine melanoma cell lines, they were able to knockdown both LDH-A and -B simultaneously and showed reduced LDH activity and lactate secretion only with double knockout (Ždralević et al., 2018).

Lactate has also been shown to trigger calcium signaling (Huang et al., 2008), angiogenesis (Fukumura et al., 2001; Shi et al., 2001; Xu et al., 2002), HIF-1α stabilization (Mekhail et al., 2004), cell death (Graham et al., 2004), suppression of anti-cancer immune response (Fischer et al., 2007) and modulation of gene expression (Moellering et al., 2008; Nowik et al., 2008; Zieker et al., 2008). All of these effects play roles in tumorigenesis. One report demonstrated that daily intra-peritoneal injections of L-lactate (500 mg/kg) into nude mice along with injection of MDA-MB-231 breast cancer cells did not further enhance primary tumour growth or vascular density, but significantly enhanced lung metastasis. The Boyden chamber assay was used to demonstrate increased MDA-MB-231 cell migration *in vitro* towards 10 mM L-lactate (Bonuccelli et al., 2010b). The direct effect of lactate on various effector functions of cancer cells remains to be studied in detail. In this report we have provided experimental evidence for the role of lactate in enhancing breast cancer cell motility, possibly in part through enhanced ERK 1/2 phosphorylation and reduced E-cadherin expression (Figure 6A). Also, LDH-A or -B knockdown in pII and YS1.2 cells reduced ERK1/2 phosphorylation (Figure 6B).

In conclusion, our data highlight the importance of targeting LDH/lactate pathway as an effective means to reduce breast cancer cell motility. This, and our previous studies, also suggest that prevailing ideas regarding the mechanisms responsible for tumour invasion and metastasis in the extracellular environment may need to be re-assessed.

## Data Availability

The raw data supporting the conclusion of this article will be made available by the authors, without undue reservation.
